# The Application of Stepwise Pelvic Devascularisation in the Management of Severe Placenta Accreta Spectrum as Part of the Soleymani and Collins Technique for Caesarean Hysterectomy: Surgical Description and Evaluation of Short- and Long-Term Outcomes

**DOI:** 10.3390/diseases13120400

**Published:** 2025-12-15

**Authors:** Hooman Soleymani majd, Lamiese Ismail, Prasanna Supramaniam, Aakriti Aggarwal, Annie E. Collins, Lee Lim, Susan Addley, Alicia Hunter, Lexie Pert, Theophilus Adu-Bredu, Pedro Pinto, Ammar Al Naimi, Jacopo Conforti, Karin Fox, Sally L. Collins

**Affiliations:** 1 Department of Gynaecology Oncology, Churchill Hospital, Oxford University Hospitals NHS Foundation Trust, Old Road, Oxford OX3 7LE, UK; 2 Nuffield Department of Women’s and Reproductive Health, University of Oxford, Oxford OX2 6GG, UK; 3 Department of Gynaecology, John Radcliffe Hospital, Oxford University Hospitals NHS Foundation Trust, Headley Way, Oxford OX3 9DU, UK; 4 Medical Sciences Division, University of Oxford, Oxford OX1 2JD, UK; 5 University Hospitals of Derby and Burton NHS Foundation Trust, Uttoxeter Road, Derby DE22 3NE, UK; 6 Serviço de Ginecologia, ULS São João Faculdade de Medicina, Universidade do Porto, 4200-427 Porto, Portugal; u010806@chsj.min-saude.pt; 7 Department of Obstetrics and Gynecology, Buergerhospital, 60318 Frankfurt, Germany; ammar.alnaimi@uclmail.net; 8 Department of Obstetrics and Perinatal Medicine, Goethe University Hospital of Frankfurt, 60596 Frankfurt, Germany; 9 Department of Clinical and Experimental Sciences, University of Brescia, 25136 Brescia, Italy; 10 Division of Maternal Fetal Medicine, Department of Obstetrics and Gynecology, Baylor College of Medicine, Houston, TX 78229, USA; 11 Birmingham Women’s Hospital, Mindelsohn Way, Birmingham B15 2TG, UK

**Keywords:** abnormally invasive placenta, interventional radiology, major obstetric haemorrhage, placenta accreta spectrum, pelvic devascularisation

## Abstract

Background: Severe (FIGO grade 3b & c) placenta accreta spectrum (PAS) is potentially a life-threatening condition due to catastrophic haemorrhage at delivery. Consequently, interventional radiology (IR) techniques are often employed to prevent massive blood loss, but this is not always readily available, is costly, and can cause significant morbidity, including distal limb ischaemia due to thrombus formation. We believe that internal iliac ligation under direct vision is a safe option to control bleeding. We sought to evaluate the short- and long-term outcomes relating to this technique compared to IR. Methods: This is a mixed-methods cohort study of women with severe PAS who underwent hysterectomy with either surgical devascularisation, as part of the Soleymani and Collins (SAC) technique, or IR insertion of internal iliac balloon catheters, in a UK tertiary referral centre for PAS between 2011 and 2022. Only women with intraoperative diagnosis of very severe PAS (FIGO stage 3b & c) were included in this study. Results: Of the 22 women invited to participate in the long-term component of the study, 59% agreed. Women in the surgical devascularisation group experienced no adverse short or late sequelae related to internal iliac arterial ligation. Pelvic devascularisation (11 patients, 41%) demonstrated a reduction in median estimated blood loss, 1600 millilitres vs. 2500 millilitres in the IR balloon catheter group (*p* = 0.04). Conclusions: We have demonstrated that the SAC technique for surgical devascularisation is a safe method for achieving haemorrhage control during caesarean hysterectomy for severe PAS. It also appears to be at least as effective at haemorrhage control as IR balloon occlusion of the internal iliac vessels.

## 1. Introduction

Severe placenta accreta spectrum (PAS), classed as the International Federation of Gynaecology and Obstetrics (FIGO) Grade 3, transforms the pelvic environment, resulting in anatomical distortion, extremely friable tissue, and increased vascularity, which is supplied by atypical sources such as the vesical and vaginal vessels [1]. Consequently, delivery carries significant risk of morbidity, usually from major haemorrhage, which often cannot be controlled by ligation of the ovarian and uterine vessels due to the abnormal collateral circulation. In its most severe form (FIGO Grade 3, previously known as percreta), PAS is associated with a 5–7% mortality rate [2,3]. Severe PAS cases are typically managed in a multidisciplinary team environment with allied specialists [4,5]. Interventional radiology (IR) to place intravascular balloons in the iliac vessels or aorta is used in many PAS centres around the world [5,6,7]. The degree of blood loss when using IR is dependent on the approach used; the more proximal the balloon placement, the more effective the intervention [8,9]. The utilisation of IR necessitates a high level of training and skill, specialised equipment, and additional time in theatre or surgery in a hybrid suite, with significant financial burden [10]. This makes surgical devascularisation under direct vision, as used in the Soleymani and Collins (SAC) technique for caesarean hysterectomy, a highly attractive alternative, especially in resource-limited settings [11].

IR, whilst effective, is not without intrinsic risks, including arterial thrombosis, aneurysm and stenosis development, vessel dissection, method failure, and neurological symptoms [10,12,13]. The current consensus from a recent literature review by Ernste et al. suggested that there is still a high prevalence of complications associated with IR (balloon occlusion) and highlighted that results from IR studies are heterogeneous [8]. Consequently, the questions being raised around the effectiveness and safety of IR have resulted in clinicians once again revisiting other options to mitigate against catastrophic haemorrhage associated with PAS.

The PAS service in our institution, a tertiary referral centre for PAS, originally involved routine use of IR. However, after several vascular complications, we advocated for a change in practice, adopting pelvic devascularisation instead. Retroperitoneal access to identify and ligate the internal iliac requires some training, which has been well established in gynaecological oncology, transplantation, and vascular surgery. It is rarely used in the obstetric setting as the surgeon must access the retroperitoneum whilst facing a large uterine body with the placenta still in situ, a manoeuvre regarded as too difficult to attempt by many. However, with the correct technique, vital pelvic vessels can be accessed and ligated under direct vision despite the presence of the enlarged uterus, thereby achieving the same vascular control and limiting IR-related complications. The effects of ligating the internal iliac vessels include significant reduction of blood flow to the anterior lower segment of the uterus, facilitating easier surgery with less risk of catastrophic haemorrhage. This remains effective until the collateral tributaries redistribute blood flow, which anecdotally occurs after 60 to 90 min in women with PAS. This potentially explains the apparent contradiction in a recent systematic review by Nabhan et al., which reported ligation of the uterine artery resulted in reduced blood loss, but ligation of the internal iliac did not [14]. Most of the included studies are not clear regarding the timing of ligation, but many describe it as ‘prophylactic’, insinuating it was performed before the hysterectomy started. Meanwhile, the uterine is not ligated until later in the surgery. As these hysterectomies are highly complex and the neo-vascularity which leads to the blood loss is usually found in the lower segment, it is very rare to reach point of greatest blood loss in under 60 min. We avoid this time constraint by preparing the vessels with an appropriately sited loose suture which is only tied if/when significant blood loss occurs later in the surgery, enabling utilisation of the full 60 min of reduced blood flow.

When treating surgically complex cases, surgeons from various disciplines often aim to provide standardised techniques, enhancing safety and reproducibility [15]. The SAC technique was our attempt to describe a standardised method for caesarean hysterectomy for severe PAS [16]. Our previous study demonstrated that these changes reduced intraoperative blood loss, intensive care unit admission, and surgical morbidity [16]. Many clinicians, however, have reservations about performing surgical devascularisation as they are unfamiliar with the technique and/or are deterred by the perceived theoretical long-term sequelae of permanently ligating the internal iliac arteries.

In this paper, we describe in more detail the method for surgical devascularisation used as part of the 25-step SAC technique for the management of severe PAS and compare both short- and long-term complications to the previous caesarean hysterectomies using IR for vascular control.

## 2. Materials and Methods

### 2.1. Method for Reducing Pelvic Blood Flow

#### 2.1.1. Surgical Devascularisation

Appropriate preparation of the vessels for ligation must be performed after the fetus is delivered but before the hysterectomy commences. This ensures that vascular control is readily available; if heavy bleeding starts, the surgeon can immediately “*turn off the tap,*” ensuring the surgical field remains visible, thereby reducing risk of accidental damage to surrounding structures.

A summary of the 10 steps for pelvic devascularisation is shown in Table 1. A detailed, step-by-step description of the full technique and figures is provided in the Supplementary Materials (File S1 and Figures S1–S6). Further details, including intra-partum photographs and videos, are also freely available for many of these steps on the OxPAT website (https://www.placentaaccretasspectrum.com).

After the 10 steps of pelvic devascularisation have been performed, the operative field is fully prepared with all relevant vessels—anterior division of the internal iliac arteries (IIAs) and common iliac arteries (CIAs) identified and prepared, in anticipation of being cross-clamped or ligated, if required. CIAs are never ligated but always slung in preparation for clamping in case of haemorrhage, while IIAs are routinely slung, ready to be ligated if required. Uterine arteries are always ligated at the origin. An intraoperative multidisciplinary team decision-making process is employed if bleeding ensues to decide if these interventions are necessary. Ligation of the internal iliac artery significantly reduces catastrophic blood flow, and clinicians should not hesitate to do this if clinically indicated.

If any inadvertent vascular injury occurs, this should also be repaired immediately. Inadvertent ligation of the external iliac artery produces an acutely ischaemic leg which classically presents with absence of distal pulses and pallor of the foot. If there is a high suspicion that the external iliac artery has been ligated, it is essential to check for a pulse in the external iliac artery above the inguinal ligament, femoral pulse in the groin, or vascular flow using an ankle Doppler. If inadvertent arterial ligation has occurred, remove the ligature. If this fails to restore a good pulse, a vascular surgeon should be called [15].

#### 2.1.2. IR Balloon Occlusion

The endovascular balloons were placed bilaterally in the IIAs using IR in the obstetric theatre prior to surgery. The woman was positioned ready for surgery so that they did not need to be moved after balloon positioning to reduce the risk of balloon migration. The balloons remained deflated until after delivery of the fetus, thereby preventing any potentially damaging reduction in placental/fetal perfusion. Following delivery, the balloons were inflated when haemorrhage control was required and removed after the surgery was completed.

### 2.2. Study of Short- and Long-Term Complications

This is a mixed-methods cohort feasibility study. Demographic and short-term outcome data were captured contemporaneously in the perioperative period and prior to hospital discharge as part of the original study [16]. National Health Service research ethical approval was obtained (22/SS/0105) to contact women regarding their long-term outcomes. The conception of the study, data analysis, interpretation, manuscript drafting, and subsequent revisions were carried out in accordance with the principles of the Declaration of Helsinki, the recommendations of the Committee on Publication Ethics, and the Reporting of studies Conducted using Observational Routinely collected Health Data (RECORD) guidelines, endorsed by the Enhancing the Quality and Transparency of Health Research Network (EQUATOR).

All patients who underwent elective treatment of PAS between January 2011 and December 2022 in our unit were considered for inclusion. Only those who had clinically confirmed very severe PAS with involvement of pelvic organs (FIGO Grade 3 b & c) at the time of caesarean hysterectomy and who had undergone prophylactic arterial balloon occlusion or surgical devascularisation were considered eligible. The cut-off of December 2022 was used to ensure only women who had been delivered more than one year before this study commenced were included; therefore, ‘long-term’ adverse outcomes in this study relate to complications apparent after a minimum of one year post surgery. The main difference between the SAC technique and our previous standard of care was the change from using IR to surgical devascularisation and from a vertical abdominal incision to a transverse one. As the technique for the hysterectomy itself remained much the same, the two groups are referred to as the IR group and the surgical devascularisation group for the rest of this paper.

Estimated blood loss was calculated by the cell salvage technician using collected blood and swab washing, combined with weighing of all theatre drapes and an estimate of spurious losses, such as blood on the theatre table and floor. The decision on when to administer blood products was taken by the anaesthetist according to unit protocols for obstetric haemorrhage. According to this, transfusion is triggered by clinical factors, such as acute haemodynamic instability and haematological findings including lactate levels and thromboelastogram (TEG) assay results. These protocols did not significantly change during the study period. Patients were only admitted to ITU if there was evidence of organ failure, such as adult respiratory distress syndrome (ARDS).

To investigate the long-term outcomes, the women were contacted by post or email, inviting them to participate. In the absence of a response after two weeks, a further letter or email was sent, but no further contact was attempted thereafter. Patients were sent a participant information sheet, consent form, and a questionnaire. The questionnaire is included in the Supplementary Materials (File S2). It was aimed at addressing complications stemming from the abdominal incision, pelvic devascularisation, and bladder reflection. Upon returning the signed consent form, a call was arranged at a mutually convenient time for questionnaire completion. The same researcher (LP) conducted all the interviews according to a pre-defined interview structure, clarifying the question if there was any difficulty in understanding it and exploring the response if there was uncertainty. At the end of the interview, the women were asked if there was anything they wished to discuss and were signposted to medical help if this revealed any issues (such as psychological distress, which is common among PAS patients). Women declining participation had their information removed and were not re-contacted.

Due to the small sample size, nonparametric testing was used with Fisher’s exact test for binary data and the Mann–Whitney test for continuous variables. Significance was set at *p* < 0.05. Analysis was conducted using the Statistical Package for Social Sciences (version 26.0; International Business Machines Corporation, Endicott, NY, USA).

## 3. Results

**Figure 1:**
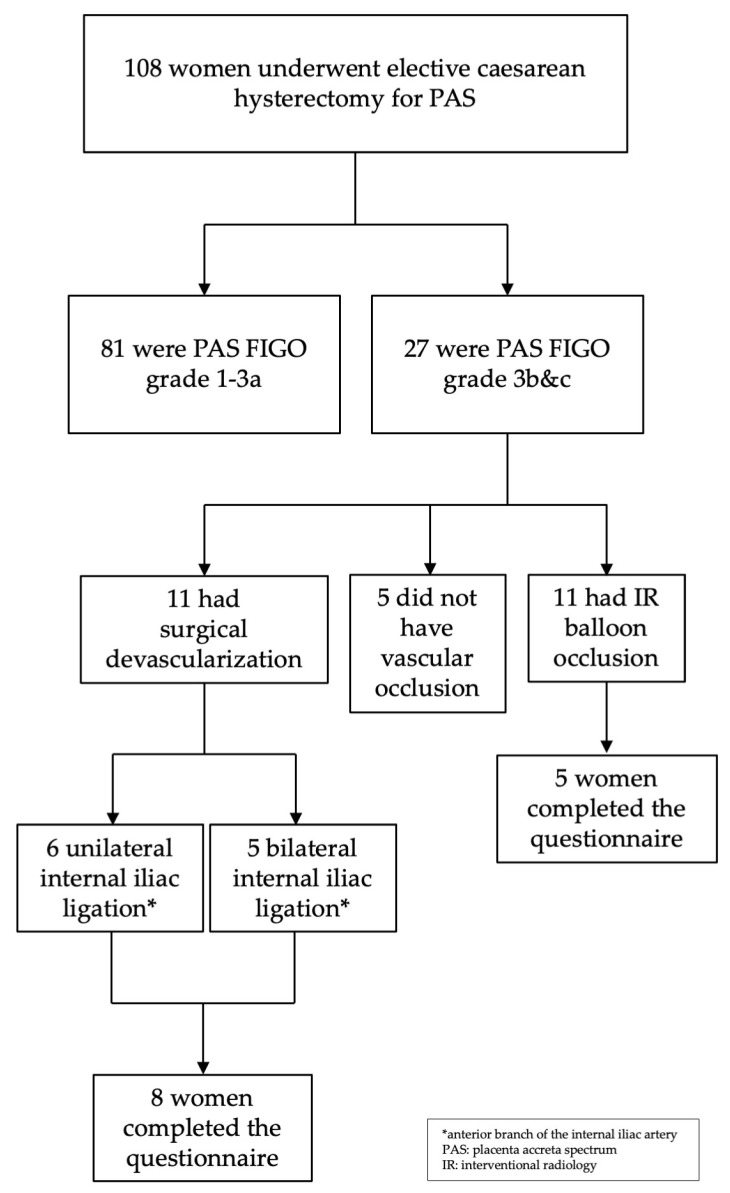
Flow chart the patient inclusion process.

Between January 2011 and December 2022, 108 women underwent elective delivery for PAS in our unit. Of those, only 22 had very severe PAS (FIGO grades 3b & c), and IR or surgical devascularisation was employed as part of their management—see Figure 1. Between 2011 and 2015, five women were not managed with IR as the service in our unit was not available due to logistical issues; hence, these women were excluded from our analysis.

### 3.1. Short-Term Complications

Eleven patients underwent IR-based haemorrhage mitigation before surgery and, therefore, were included in this analysis of short-term complications. Eleven women had surgical devascularisation consisting of ligating the uterine arteries at their origin from the IIA, while the decision to occlude the IIA itself was made intraoperatively according to the degree of bleeding. The presence and laterality of parametrial invasion guided whether unilateral or bilateral IIA ligation was required.

Table 2 contains data on the patient demographics, surgical techniques, and intra- and perioperative (short-term) morbidity for the 22 women.

There were no statistically significant differences seen in age at delivery, body mass index, or gestation at delivery. The median estimated blood loss in the pelvic devascularisation group was 1600 millilitres (mL) vs. 2500 mL in the IR group (with an inter-quartile range of 1135 mL and 2050 mL, respectively). There was no statistically significant difference when comparing blood transfusion between the groups. In the IR group, one woman had a catastrophic haemorrhage requiring transfusion of >10 units of packed red cells and admission to the intensive care unit, with adult respiratory distress syndrome.

### 3.2. Long-Term Complications

In the long-term follow-up group (*n* = 13), none of the women in either group reported symptoms of ischaemic leg pain or buttock claudication (Table 3). Two women, one in each group, reported transient tingling and/or numbness in one or both legs, which had subsequently resolved. Two women in the surgical devascularisation group responded “yes” when asked about other leg symptoms, but on further questioning, these symptoms were unlikely to be related to their surgery; one had recently been formally diagnosed with arthritis, and the other described generalised stiffness, which was eased by movement. Although not on the questionnaire, during the interview, one woman in the IR group revealed she had developed pelvic pain after the surgery, which she reported was diagnosed as “*a narrowing of a blood vessel*”. She reported she had undergone endovascular dilatation, which had mostly resolved the symptoms. We were unable to obtain medical records to confirm this.

## 4. Discussion

This study demonstrates that clinically indicated surgical devascularisation for very severe, potentially life-threatening PAS is safe and appears to be at least as effective as the commonly employed IR balloon occlusion of the internal iliacs in the prevention of catastrophic haemorrhage. As well as reducing cost and operative time/logistic challenges, surgical ligation of the IIA under direct vision has the advantage of ensuring precise placement of the occlusion and prevents endovascular damage from arterial catheterisation. Once the technique has been learned by the surgeon, it can also be employed in the event of unanticipated haemorrhage, while IR cannot.

We acknowledge that by only selecting the very worst cases in an already rare condition, the sample size is, by default, small. However, PAS is a spectrum with the majority of cases being mild to moderate which, with appropriate MDT management, should not require haemorrhage control strategies that carry substantial risk of iatrogenic morbidity. The small sample size ensures homogeneity but reduces the power to detect rare risks, and means any inference from statistical significance is very limited. The statistical significance observed in estimated blood loss, whilst encouraging, should be interpreted with caution as the large interquartile ranges suggest substantial variability. However, the purpose of the study was to demonstrate that, in severe PAS, ligation of the internal iliacs is a safe option for haemorrhage control. If adverse outcomes associated with either vascular occlusion technique were truly frequent, we would have expected to observe them t despite the small sample size.

By evaluating patient outcomes following pelvic devascularisation in the obstetric setting, we have demonstrated its safety, feasibility, and efficacy for the treatment of severe PAS. Our patient cohort experienced no adverse immediate or late sequelae despite having the anterior division of internal iliac arteries ligated (unilaterally or bilaterally) as part of comprehensive pelvic devascularisation. There has been no apparent morbidity demonstrated in this group despite a long-term follow-up period of at least 1 year. Given the perception by many obstetricians that ligation of the internal iliac arteries will lead to such severe morbidity that it should not be undertaken even in the event of potentially life-threatening haemorrhage, we believe these results are important.

The authors recognise that another limitation of the study is that long-term outcomes were assessed as part of a telephone follow-up, so there may be some recollection bias from patients who had longer surgery-to-questionnaire intervals. However, the magnitude of bias is likely to be small, as one would expect patients to recall significant occurrences related to vascular complications, as these would likely have affected the patient’s quality of life (such as buttock claudication). The strengths of this study include the fact that it was conducted in one centre, by the same team, in a consistent and reproducible manner, achieving the same results.

Care must also be taken when interpreting the short-term outcomes. The study included all eligible patients during a set time period. Whilst the surgical team remained the same during the time period, the change from IR to surgical devascularisation occurred in our team in 2016; the statistical difference seen in blood loss and blood products between the two groups may be a result of temporal confounding from increased experience—a learning curve effect. The purpose of this study was to investigate the inherent safety of surgical devascularisation rather than efficacy in comparison to IR. However, it is encouraging that, despite the small sample size, surgical devascularisation appears to be as effective as the widely used IR balloon occlusion.

We believe the timing of the occlusion of the internal iliacs is vitally important due to the massive collateral circulation seen in PAS. Previous studies of prophylactic IIA ligation before the surgery commences have failed to demonstrate significant haemorrhage control. This is potentially due to the relatively rapid opening of the collateral circulation after IIa ligation (anecdotally 60–90 min), negating the vascular control strategy. Conversely, this may not be seen with balloon occlusion as many strategies involve regularly deflating the balloons at a set period of time (usually every 15 to 30 min), decreasing the likelihood of the collateral vessels being recruited in a relatively short period of time. We would encourage PAS surgeons to prepare for vascular occlusion either surgically or with IR, but only employ it in the face of actual haemorrhage occurring.

We have described a method of stepwise pelvic devascularisation and demonstrated the feasibility, safety, and efficacy of preparing the internal iliac arteries to be ligated in case of haemorrhage, in order to “*turn off the tap*”. As noted by other non-obstetric studies, we did not find an increased incidence of immediate or delayed complications such as urinary or gastrointestinal tract injury, bladder atony, gluteal necrosis, or buttock claudication in women undergoing internal iliac artery ligation for severe PAS [17]. This is due to a systematic approach involving direct visualisation of the anterior division of the internal iliac artery.

## 5. Conclusions

We envisage that this paper will benefit patients by building clinicians’ knowledge and confidence in performing pelvic devascularisation when required for control of significant haemorrhage. Even though this technique has been described in the context of severe PAS, it may be life-saving for any type of complex pelvic surgery or major pelvic haemorrhage. This study demonstrates that the surgical approach to reducing vascular bleeding is feasible in an obstetric setting despite the presence of a significantly enlarged uterus. We therefore suggest instituting an obstetric training programme to teach pelvic devascularisation for the management of severe haemorrhage when IR is not available.

## Figures and Tables

**Table 1:** Summary of the steps for pelvic surgical devascularisation.

Step	Description of the SAC Surgical Devascularisation Technique
1	Fundal caesarean delivery with placenta left in situ and hysterotomy closed
2	Access to the abdominal aorta below the inferior mesenteric artery, with exposure of the aorto-caval region
3	Exposure and identification of the right common iliac artery and bifurcation, IVC, and right ureter
4	Identification of the aorta-caval space, followed by slinging the right common iliac artery
5	Suture double-tied but left loose enough not to occlude blood flow, placed at the correct position on the right internal iliac artery (ligation can then instantly be performed by tightening the knots)
6	Ligation of the right uterine artery at its origin
7	Exposure and identification of the left common iliac artery, left common iliac vein and left ureter
8	Identification and slinging of the left common iliac artery
9	Suture double-tied but left loose enough not to occlude blood flow, placed at the correct position on the left internal iliac artery (ligation can then instantly be performed by tightening the knots)
10	Ligation of the left uterine artery from the origin

**Table 2:** Summary of patient demographics, surgical techniques employed, and short-term maternal morbidity for all 22 patients.

Patient Demographics	Surgical Devascularisation (*n* = 11)	IR (*n* = 11)	*p* Value
Age at the time of surgery	33 (5.7)	35 (3.1)	0.24 *
Body mass index at the time of surgery	26.1 (5.6)	25.8 (8.5)	0.28 *
Gestation at delivery (completed weeks)	35.5 (1.7)	35 (0.8)	1 *
Devascularisation Technique			
Internal iliac artery ligation (unilateral)	6 (55%)	0	
Internal iliac artery ligation (bilateral)	5 (45%)	0	
Pelvic arterial balloon placement (IR)	0	11	
Maternal Morbidity			
Median estimated blood loss (with interquartile range)	1600 (1135) mL	2500 (2050) mL	0.04 * (r_b_ = 0.45)
Received blood products	2 (18%)	7 (63.6%)	0.08 ^†^
Significant transfusion (>5 units red cells)	0	3	0.21 ^†^
Massive transfusion (>10 units red cells)	0	1	0.59 ^†^
Intensive care unit admission with adult respiratory distress syndrome	0	1	0.59 ^†^

*p* values calculated using * the Mann–Whitney U test, with results given as median (IQR) and *p* value with rank biserial correlation (r_b_), and ^†^ Fisher’s exact test.

**Table 3:** Long-term outcomes—patient-reported signs and symptoms experienced at any point since the surgery.

	Surgical Devascularisation	IR	*p* Value
Number of patients contacted	11	11	-
Number of patients responded	8	9	-
Number of patients who declined to participate	0	4	-
Number of study participants	8 (73%)	5 (45%)	-
	*n* = 8	*n* = 5	
Time since surgery in years	2.5 (4)	11 (2.5)	0.02 *
Ischaemic leg pain	0	0	-
Transient tingling/numbness in leg	1 (12.5%)	1 (20%)	0.64
Buttock claudication	0	0	-
Other leg symptoms (see text)	2 (25%)	0	0.36
Inability to open bowels/pass wind	0	0	-
Bladder symptoms requiring urodynamic testing	0	1	0.38
Satisfaction with the results of the surgery	7 (88%)	4 (80%)	1.00

*p* values calculated with Fisher’s exact test, except *, which was calculated with Mann–Whitney U test; results given as median values (IQR).

## Data Availability

Data are only available under reasonable request directly to the corresponding author to protect the privacy of the patients.

## Supplementary Materials

The following supporting information can be downloaded at: https://www.mdpi.com/article/10.3390/diseases13120400/s1, Figure S1: shows aorta bifurcation and inferior vena cava and their relationship with the ureter; Figure S2 illustrates how to sling the IIA; Figure S3 shows how to sling the left CIA; Figure S4 shows how to sling the right CIA; Figure S5 moment when the common iliac artery is slung; Figure S6 ligation of the uterine artery at its origin; File S1 description of the surgical steps for the SAC procedure; File S2 questionnaire administrate to patients.

## Abbreviations

The following abbreviations are used in this manuscript:

PAS	Placenta Accreta Spectrum
SAC	Soleymani and Collins
IR	Interventional Radiology
CIA	Common Iliac Artery
IIA	Internal Iliac Artery
