# Supplementary material for: The Application of Stepwise Pelvic Devascularisation in the Management of Severe Placenta Accreta Spectrum as Part of the Soleymani and Collins Technique for Caesarean Hysterectomy: Surgical Description and Evaluation of Short- and Long-Term Outcomes
Source: Diseases. 2025 Dec 15;13(12):400. doi: 10.3390/diseases13120400 (PMC12731549; doi:10.3390/diseases13120400)
Supplement: Supplementary file 1 [file diseases-13-00400-s001.zip › diseases-4028271 File S2 questionnaire.pdf]

Questionnaire Questions

"Since your surgery………"	Yes	No	N/A
Have you noticed reduced or altered sensation in your groin or inner thigh?			
Have you noticed burning pain in the lower abdomen?			
Have you noticed reduced or altered sensation in the front of the thigh (below the groin crease)?			
Have you been unable to open your bowels or pass wind; associated with abdominal pain, nausea, and/or vomiting?			
Do you have any bulging or protruding tissue under the skin of the scar from the surgery?<br>*If yes:*<br>*Did you need to have any surgery for this? Have you had any complications from the repair?*			
Any swelling or collection of fluid under the skin of the scar from the surgery?<br>*If yes:*<br>*Have you had any scans for this?*			

On a scale from 1-10, please rate the following:	Enter number (1-10):
How satisfied are you about the cosmetic appearance of your scar? (1= extremely dissatisfied, 10= extremely satisfied)	
Is the scar painful? (1= not at all, 10= yes very much)	
Is the scar itchy?	
Is the colour of the scar different from your normal skin?	
Is the stiffness of the scar different from your normal skin?	
Is the thickness of the scar different from your normal skin?	
Is the scar more irregular your normal skin?	

	Yes	No	N/A
Do you feel your mental health been negatively affected by the surgery?			
Have you been diagnosed with PTSD since having the surgery?			
Are you satisfied with the outcome of the surgery?			
Have you had any leg pain that is brought on by exercise and relieved by rest?			
Have you had any tingling/numbness in your leg, or has your leg been cold, painful or appear mottled?			
Have you had any buttock pain?			
Have you had any:<br>- *Shooting pain down the back of the leg?*<br>- *Numbness or loss of sensation over the skin of your leg?*<br>- *Any tingling, burning or pins and needles in your leg?*<br>- *Any weakness in your leg muscles?*			
	**Yes**	**No**	**N/A**
Have you had an appointment for a 'urodynamics' test, to check the function of your bladder / waterworks?			
Have you had any involuntary urine leakage from your vagina, which required investigation?			
