# Supplementary material for: The Application of Stepwise Pelvic Devascularisation in the Management of Severe Placenta Accreta Spectrum as Part of the Soleymani and Collins Technique for Caesarean Hysterectomy: Surgical Description and Evaluation of Short- and Long-Term Outcomes
Source: Diseases. 2025 Dec 15;13(12):400. doi: 10.3390/diseases13120400 (PMC12731549; doi:10.3390/diseases13120400)
Supplement: Supplementary file 1 [file diseases-13-00400-s001.zip › diseases-4028271- File S1.pdf]

The SAC technique for pelvic devascularisation

STEP 1: Abdominal entry

Positioning the patient in modified Lloyd Davis, enables vaginal access for cervical manipulation during hysterectomy and facilitates easy assessment of any vaginal blood loss, whilst avoiding well-leg compartment syndrome and femoral nerve neuropraxia. The abdominal incision is decided pre-operatively according to the patient's body habitus, history and preference but is typically done using the Soleymani and Collins transverse incision. PAS diagnosis is confirmed, the uterus and fetus delivered and the hysterotomy closed.

STEP 2: Access to the abdominal aorta below the inferior mesenteric artery, with exposure of the aorto-caval region

Following division of the round ligament, access to the retroperitoneal spaces, abdominal aorta, common iliac artery, inferior vena cava (IVC), and pelvic vasculature is achieved by mobilizing the ascending colon along the Toldt line (the lateral peritoneal reflection along the outer edge of the ascending and descending colon), up to the hepatic flexure (Figure 1). This can be done using atraumatic forceps (Roberts dissecting forceps) and monopolar diathermy to ensure fine dissection. This is extrapolated from the Cattle-Braasch manoeuvre (right-sided medial visceral rotation) allowing full access to the right para-colic and pelvic side wall after identification of the abdominal aorta below the inferior mesenteric artery. The ascending colon is then packed to the level of hepatic flexure using malleable retractors covered with wet swabs to prevent damage. It is good practice to release and re-apply these self-retaining retractors every 30-40 minutes during surgery to prevent femoral nerve neuropraxia.

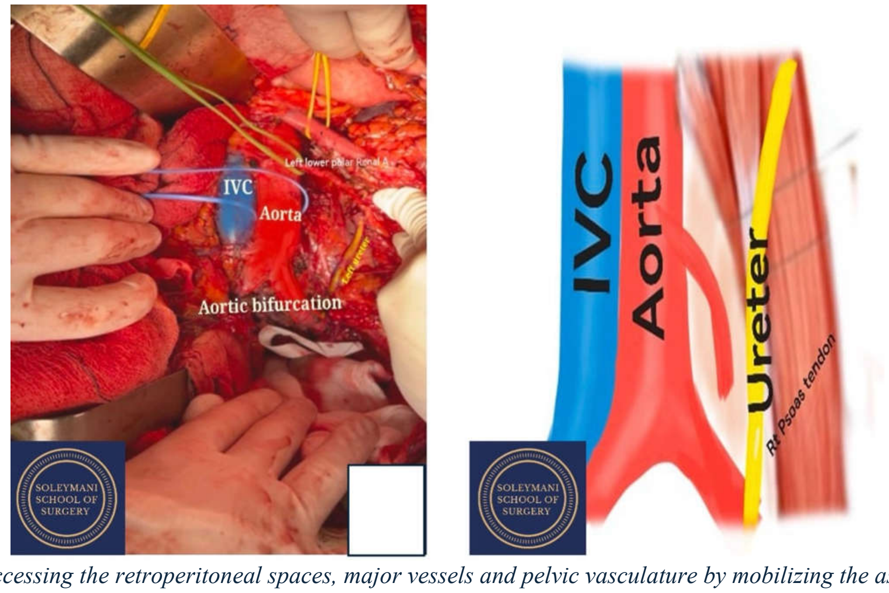

Figure S1 Accessing the retroperitoneal spaces, major vessels and pelvic vasculature by mobilizing the ascending colon along the Toldt line

STEP 3: Exposure and identification of the right common iliac artery (RCIA) and bifurcation, IVC and right ureter

Once the pelvic side wall has been opened, the RCIA with its bifurcation and IVC can be readily identified. The broad ligament has already been opened as part of the previous manoeuvre. The

operating surgeon applies their non-dominant hand to medialise the posterior leaf of the broad ligament. The right ureter can now be visualised medially running parallel to the right gonadal vessels. The ureter can be slung carefully using a vessel loop (Figure 2). The right ureter is grasped with Russian forceps while introducing a long right-angled dissecting forceps (eg. O' Shaugnessy) underneath the ureter (typically from lateral to medial). The assistant passes a vessel loop into the slightly opened jaws of the forceps. The end of the sling is secured with small artery forceps, taking care that the ureter is not under tension. Further medialisation of the uterine body and gonadal vessel will expose and develop the roof, lateral and medial borders of the right para-rectal space, exposing the entire RCIA.

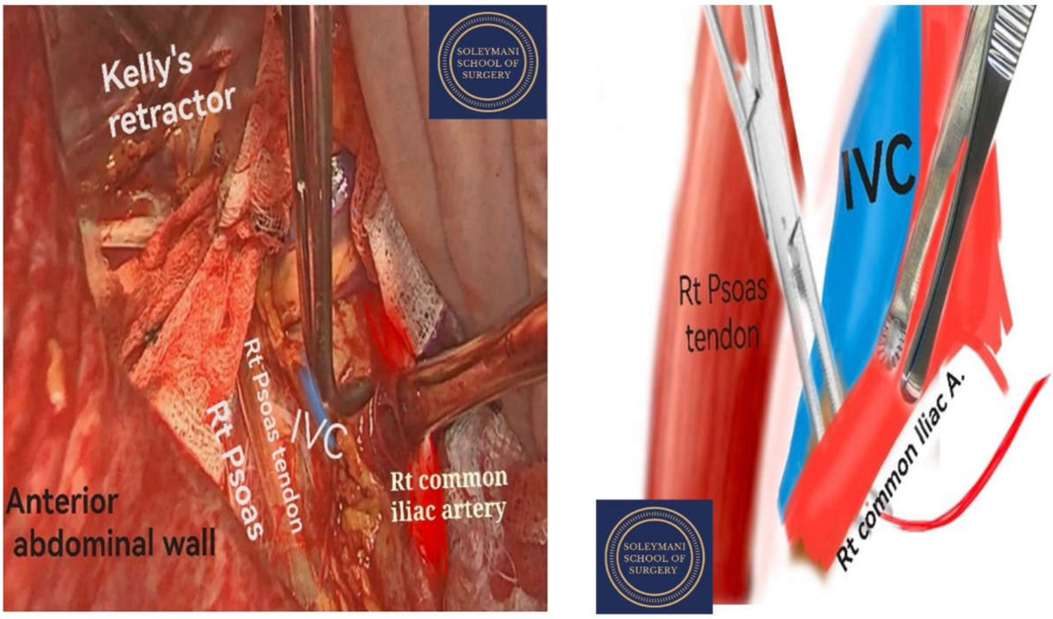

Figure S2 The right ureter can now be slung, it is visualised medially, running parallel to the right gonadal vessels

<u>STEP 4: Identification of aorta-caval space followed by slinging the right common iliac artery (RCIA)</u>

The assistant applies a malleable retractor lateral to the ureter, medialising this further to keep the space open and free-up the hands of the operating surgeon to carefully grasp the RCIA. A vessel loop is placed below the RCIA whilst cognisant of its proximity to the IVC. The IVC lies posterolateral to the RCIA and therefore when slinging the RCIA, the surgeon needs to go from lateral to medial to avoid inadvertently injuring the IVC. To sling the RCIA, use Russian forceps to carefully grasp the RCIA and introduce a long right-angled forceps (Lahey or O' Shaugnessy) from lateral to medial, while the assistant passes a vessel loop so that the RCIA sling is in place. This is in preparation for the potential application of a Fogarty or De Bakey vascular clamp in the event of catastrophic haemorrhage.

<u>STEP 5: Slinging +/- ligation of the right internal iliac artery (RIIA)</u>

This requires further development of the avascular spaces of right pelvic side wall, including lateral pararectal and para-vesical spaces. The lateral retroperitoneal spaces lie next to the rectum

bilaterally (commonly referred to as the pararectal spaces). The pararectal spaces are divided by the ureter into the medial (Okabayashi space) and lateral para-rectal space (Latzko space).

The surgeon then further dissects the pararectal space, parallel and medial to the ureter where the inferior hypogastric plexus lies. This nerve plexus innervates the bladder, and it is important to avoid injuring it. The surgeon should now have access to the right external and internal iliac arteries and veins. To sling the RIIA, the contents of the pararectal space are medialized using a malleable retractor. This allows the operating surgeon to sling the RIIA as described for the RCIA using a Russian forceps and right-angled dissecting forceps (applied lateral to medial). The surgeon uses a 1-0 tie or sling around the anterior division of the RIIA 3-3.5cm distal to the bifurcation (Figure 3). This exact placement is required to avoid the posterior division and its branches (namely the superior gluteal, lateral sacral and ilio-lumbar arteries). The posterior division usually comes off almost immediately after the bifurcation, so tying it too close to the bifurcation would increase the risk of inadvertently tying off these vessels.

Care should be taken to avoid injury to the internal iliac vein (IIV), which lies in proximity on the postero-medial aspect of the internal iliac artery (IIA). So, when grasping the anterior division of the IIA and applying the angled clamp, the tip of the clamp should be elevated off the tissue to avoid injuring the IIV. This is very important as any bleeding from the IIV could be challenging to control.

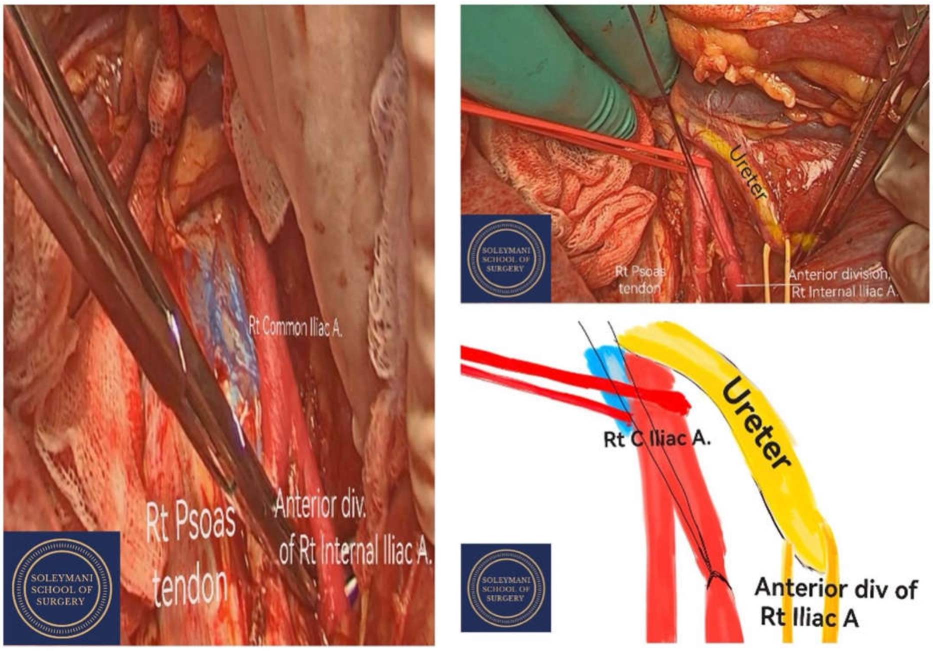

Figure S3 Ligation of the anterior branch of internal iliac artery

<u>STEP 6: Division of the right uterine artery from the origin</u>

Dissection in the lateral pararectal space, will result in exposure of the right uterine artery. Further dissection of the paravesical space (retroperitoneal space that lies lateral to the urinary bladder, anterior and superior to the pararectal space) will help adequate exposure of the uterine artery and its origin from the anterior division of the RIIA. Next the operating surgeon performs ligation

and division of the right uterine artery at the origin, using either medium or large titanium ligating clips (Ligaclips) and/or 0 vicryl ties.

STEP 7: Exposure and identification of the left common iliac artery (LCIA), left common iliac vein and left ureter

The procedure is then repeated on the contralateral side by mobilization the descending colon to the level of splenic flexure. This is extrapolated from Mattox manoeuvre (left medial visceral rotation), to expose and identify the LCIA and left common iliac vein (situated to the medial aspect of the LCIA) (Figure 4).

The operating surgeon applies their non-dominant hand to medialise the posterior leaf of the broad ligament and identify the left ureter (running parallel to the left gonadal vessels). This is followed by grasping the left ureter with Russian forceps and using a long right angle dissecting forceps to pass a vessel loop underneath it. Further medialisation of the uterine body, and gonadal vessel exposes and develops the roof, and lateral and medial borders of the left para-rectal space exposing the LCIA in its entirety.

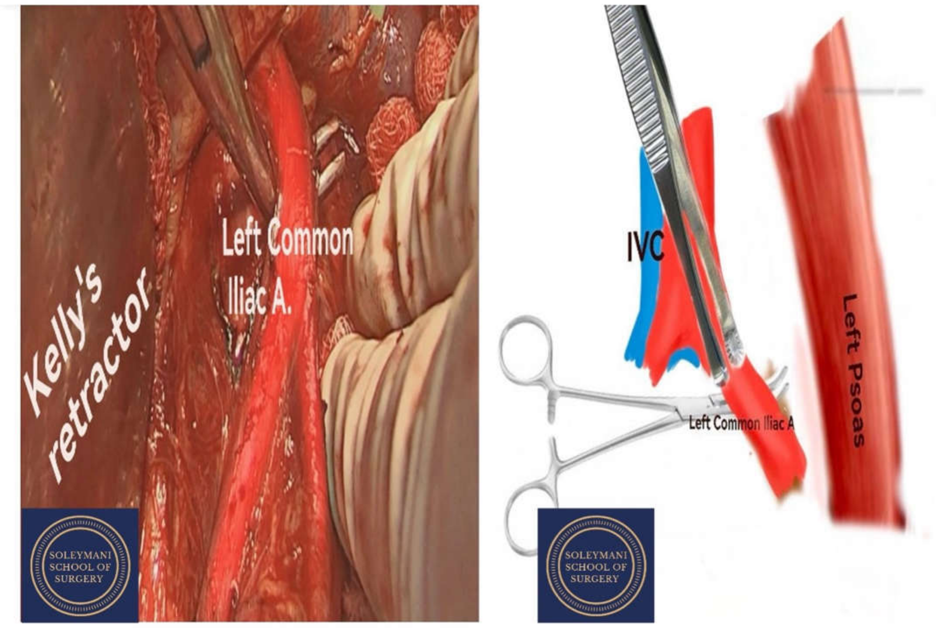

Figure S4 Exposure of the left common iliac artery

STEP 8: Identification and slinging of the left common iliac artery

The assistant applies a malleable retractor lateral to the ureter and medialises the tissues further to keep this space open, freeing-up the hands of the operating surgeon to grasp the LCIA and pass a vessel loop as previously described for the RCIA. However, as the left common iliac vein lies posteromedial to the artery, the long right-angled dissecting forceps is inserted medial to lateral, with the tip carefully pointing upwards on the way out (Figure 5).

Again, the surgeon should carefully check for any inadvertent injury to iliolumbar and median sacral veins emptying to the left common iliac vein. It should be noted that if vascular clamps have

been applied to the common iliac artery, careful time keeping on these clamps is crucial, as they should be released after 60 minutes of occlusion.

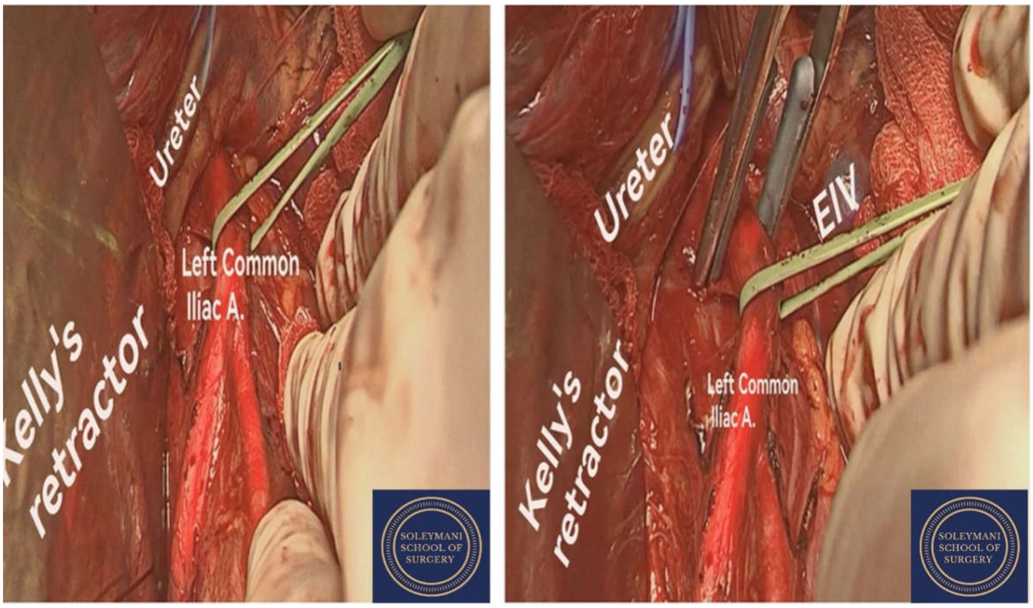

Figure S5 The left common iliac artery is now slung

STEP 9: Slinging +/- ligation of the left internal iliac artery

Further development of the avascular spaces of left pelvic side wall, including lateral pararectal and para-vesical spaces occurs, as described for the right side. The surgeon now has access to the left external and internal iliac arteries and veins. To sling the left internal iliac artery, the contents of the pararectal space are medialized using a malleable retractor. This allows the operating surgeon to sling the left internal iliac artery as described previously using a Russian forceps and a long right-angled dissecting forceps, applied from lateral to medial. Again a 1-0 tie is used 3-3.5cm distal to the bifurcation of the LCIA, being careful to avoid injury to the left internal iliac vein.

STEP 10: Division of the left uterine artery from the origin

This is followed by exposure of left uterine artery after development of para-vesical space and division of the left uterine artery from the origin using medium or large titanium ligation clips (Ligaclips) and/or 0 vicryl ties (Figure 6).

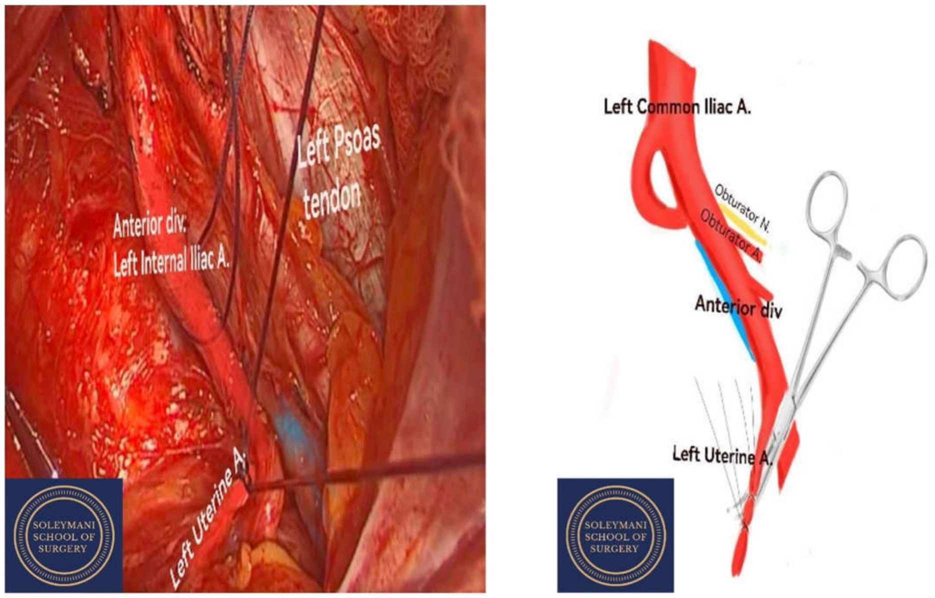

Figure S6 The uterine artery is clipped
